# Supplementary material for: Prioritization of the concepts and skills in quantitative education for graduate students in biomedical science
Source: PLoS One. 2023 Apr 27;18(4):e0284982. doi: 10.1371/journal.pone.0284982 (PMC10138463; doi:10.1371/journal.pone.0284982)
Supplement: Attachment — Submitted filename: PLOS review_11282022.docx [file pone.0284982.s004.docx]

Summary:

The article entitled Prioritization of the concepts and skills in quantitative education for graduate students in biomedical science elevates a long-standing question of aligning graduate student preparedness with program-based curricular. This work situates the current/historical perspectives of how program curriculum may vary by school, and department levels, and the implications of graduate student readiness and/or requirement for tailored curriculum. The novel approach of using faculty survey of literature and identifying preferential requisites necessary for graduate students in their prospective fields offers and opportunity for best practices in curriculum design in the biomedical sciences. Overall and very nice article worthy of publication.

Minor comments:

Methodology and Statistics: The methodology implemented was well-designed and sample size and statistics used were of strong rigor.

Results: Results are well present allowing for clear interpretation of author’s findings including supplemental data shown.

Minor concern: The reviewer would appreciate further discussion as it relates to how alignment of student needs locally can be expanded to discipline across institutions.
